# Improved benefit of continuing luspatercept therapy: sub-analysis of patients with lower-risk MDS in the MEDALIST study

**DOI:** 10.1007/s00277-022-05071-8

**Published:** 2023-01-13

**Authors:** Ulrich Germing, Pierre Fenaux, Uwe Platzbecker, Rena Buckstein, Valeria Santini, María Díez-Campelo, Aylin Yucel, Derek Tang, Shannon Fabre, George Zhang, Roberto Zoffoli, Xianwei Ha, Dimana Miteva, Christina Hughes, Rami S. Komrokji, Amer M. Zeidan, Guillermo Garcia-Manero

**Affiliations:** 1 University Clinic, Department of Hematology, Oncology and Clinical Immunology, Heinrich-Heine University, Düsseldorf, Germany; 2 Service d’Hématologie Séniors, Hôpital Saint-Louis, Université Paris, Paris, France; 3 Hematology and Cellular Therapy, Medical Clinic and Policlinic 1, University Hospital Leipzig, Leipzig, Germany; 4 Odette Cancer Centre, Sunnybrook Health Sciences Centre, Toronto, ON Canada; 5 MDS Unit, Azienda Ospedaliero Universitaria Careggi, University of Florence, Florence, Italy; 6 Hematology Department, Institute of Biomedical Research of Salamanca, University Hospital of Salamanca, Salamanca, Spain; 7 Bristol Myers Squibb, Princeton, NJ USA; 8 Bristol Myers Squibb, Braine-L’Alleud, Belgium; 9 Moffitt Cancer Center, Tampa, FL USA; 10 Department of Internal Medicine, Yale University, New Haven, CT USA; 11 Yale Cancer Center, New Haven, CT USA; 12 Department of Leukemia, The University of Texas MD Anderson Cancer Center, Houston, TX USA

**Keywords:** Myelodysplastic syndromes, Anemia, Luspatercept, Red blood cell transfusion independence, Health-related quality of life

## Abstract

Red blood cell transfusion independence (RBC-TI) is an important goal in treating lower-risk myelodysplastic syndromes with ring sideroblasts. In the phase 3 MEDALIST study, RBC-TI of ≥ 8 weeks was achieved by significantly more luspatercept- versus placebo-treated patients in the first 24 weeks of treatment. In this post hoc analysis, we evaluated RBC transfusion units and visits based on patients’ baseline transfusion burden level and the clinical benefit of luspatercept treatment beyond week 25 in initial luspatercept nonresponders (patients who did not achieve RBC-TI ≥ 8 weeks by week 25) but continued luspatercept up to 144 weeks. RBC transfusion burden, erythroid response, serum ferritin levels, and hemoglobin levels relative to baseline were evaluated. Through week 25, fewer RBC transfusion units and visits were observed in luspatercept-treated patients versus placebo, regardless of baseline transfusion burden. This continued through 144 weeks of luspatercept treatment, particularly in patients with low baseline transfusion burden. Sixty-eight patients were initial nonresponders at week 25 but continued treatment; most (81%) received the maximum dose of luspatercept (1.75 mg/kg). Sixteen percent achieved RBC-TI for ≥ 8 weeks during weeks 25–48, 26% had reduced RBC transfusion burden, 10% achieved an erythroid response, 44% had reduced serum ferritin, and hemoglobin levels increased an average of 1.3 g/dL from baseline. These data have implications for clinical practice, as transfusion units and visits are less in luspatercept-treated patients through week 25 regardless of baseline transfusion burden, and continuing luspatercept beyond week 25 can potentially provide additional clinical benefits for initial nonresponders. Trial registration: NCT02631070.

**Supplementary Information:**

The online version contains supplementary material available at 10.1007/s00277-022-05071-8.

## Introduction

Myelodysplastic syndromes (MDS) are a heterogenous group of clonal bone marrow disorders characterized by inadequate hematopoiesis; peripheral cytopenias, particularly anemia; and an increased risk of progression to acute myeloid leukemia [1, 2]. The majority of patients with MDS fall into the lower-risk (LR)-MDS category, defined as having a risk of Very low, Low, or Intermediate disease according to the Revised International Prognostic Scoring System (IPSS-R) [3] with a score of ≤ 3.5 points [3–5]. Chronic anemia is the predominant cause of morbidity and quality of life impairment in patients with LR-MDS; a major goal of treatment is to manage anemia and its associated complications. Most patients will develop anemia at some point and will become dependent on red blood cell (RBC) transfusions, which carries the risk of iron overload and associated sequelae [6, 7]. Iron overload resulting from RBC transfusions is an independent, adverse prognostic factor for overall survival (OS) and leukemia-free survival in patients with MDS [8], significantly worsening the survival of patients with MDS with refractory anemia [6].

Real-world, observational studies have shown an inverse relationship between the burden of ongoing RBC transfusion and survival in patients with anemia due to LR-MDS, even among those with low initial RBC transfusion burden [9, 10]. Other reports have linked dependency on RBC transfusions to significantly shorter OS and leukemia-free survival [6, 11–14], more severe disease [15], and reduced health-related quality of life [14, 16] than observed in patients with RBC transfusion independence (RBC-TI). Transfusion dose density has also been shown to be inversely associated with progression-free survival, indicating that even at relatively low dose densities, reliance on transfusions is an indicator of inferior progression-free survival [9].

Eliminating or reducing the need for RBC transfusions could improve the overall health-related quality of life of patients with LR-MDS while also reducing health care resource use and associated costs. However, very few therapeutic options are available to these patients after failure of erythropoiesis-stimulating agents (ESAs). Alternative treatments are a priority, one of which is luspatercept, a first-in-class recombinant fusion protein that binds select transforming growth factor β superfamily ligands thus inhibiting downstream Smad2/3 signaling, which enables late-stage erythroid blast differentiation and erythroid maturation [17–19]. In the phase 3 MEDALIST study (NCT02631070), luspatercept treatment resulted in RBC-TI for 8 weeks or longer in 38% of patients treated with luspatercept versus 13% of patients treated with placebo by week 25 (*p* < 0.001) [20]. Erythroid responses with associated increases in hemoglobin levels were also achieved in a greater proportion of patients treated with luspatercept than placebo (53% vs 12%), demonstrating a reduction in the severity of anemia in these patients [20]. Based on these findings, luspatercept is now approved in the USA, Canada, and Europe to treat anemia resulting from LR-MDS and ring sideroblasts [21–23].

Translating the findings from the MEDALIST study to clinical and health policy decision-making requires broader considerations of real-world patients and clinical scenarios. It is important to understand how luspatercept may reduce RBC transfusion burden relative to the patient’s level of transfusion burden before initiation of treatment. In addition, the potential clinical benefits of continuing luspatercept treatment beyond week 25, with the aim of achieving RBC-TI or substantially reducing anemia burden (for those who remained transfusion dependent at week 25), are not well understood and would be relevant to clinicians to help guide treatment decision-making.

In this post hoc analysis of MEDALIST, we evaluated the impact of luspatercept on cumulative RBC transfusion units and RBC transfusion visits over time for treated patients based on their pretreatment transfusion burden levels. Furthermore, we evaluated the clinical benefits of continuing luspatercept treatment beyond week 25 in patients who had not achieved RBC-TI for ≥ 8 weeks during the first 24 weeks of MEDALIST.

## Methods

### Study design

The design of the MEDALIST study has been described previously [20]. Briefly, eligible patients were ≥ 18 years of age; had LR-MDS according to IPSS-R criteria with ring sideroblasts; were refractory, intolerant, or unlikely to respond to ESAs; and had required regular RBC transfusions (≥ 2 units/8 weeks) in the 16 weeks prior to randomization.

**Fig. 1:**
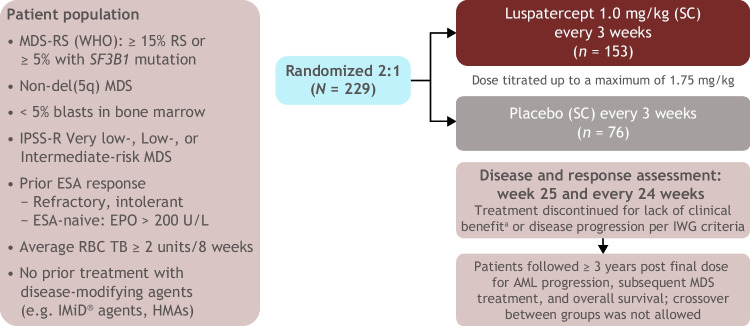
MEDALIST study design. ^a^Investigator’s assessment of clinical benefit: achievement of RBC-TI ≥ 8 weeks and/or HI-E response during weeks 1–24. AML, acute myeloid leukemia; EPO, erythropoietin; ESA, erythropoiesis-stimulating agent; HI-E, hematologic improvement-erythroid; HMA, hypomethylating agent; IMiD, immunomodulatory imide drug; IPSS-R, Revised International Prognostic Scoring System; IWG, International Working Group; MDS, myeloid dysplastic syndromes; RBC, red blood cell; RBC-TI, red blood cell transfusion independence; RS, ring sideroblasts; SC, subcutaneous; *SF3B1*, splicing factor 3B subunit 1; WHO, World Health Organization

Patients were randomized 2:1 to receive either luspatercept 1.0 mg/kg subcutaneously or placebo every 3 weeks (Fig. 1). Patients could receive luspatercept dose adjustments during the trial (up to a maximum of 1.75 mg/kg) [20]. The primary efficacy endpoint of MEDALIST was the achievement of RBC-TI for ≥ 8 weeks during the primary treatment phase (weeks 1–24). This was evaluated at week 25, defined as 24 calendar weeks after the first dose, regardless of any dose delays.

Regardless of meeting the primary endpoint at week 25, patients who had shown clinical benefit (as assessed by the investigators) without disease progression (according to International Working Group (IWG)-MDS criteria) [24] could continue to receive luspatercept or placebo in a double-blind treatment extension phase until no clinical benefit, disease progression, unacceptable side effects, or withdrawal from the study. The extension phase continued with 3-week treatment cycles.

### Assessments

This post hoc analysis of MEDALIST evaluated the cumulative mean number of RBC transfusion units and RBC transfusion visits through 144 weeks of treatment according to baseline RBC transfusion burden level (low vs high). Low and high baseline RBC transfusion burden levels were defined as having < 6 RBC transfusion units or ≥ 6 RBC transfusion units over 8 weeks, respectively. The cumulative mean number of RBC transfusion units and RBC transfusion visits required during the treatment period according to baseline RBC transfusion burden level was also evaluated by luspatercept response at week 25. Patients in the luspatercept arm who achieved RBC-TI for ≥ 8 weeks by week 25 were defined as luspatercept responders; those who did not were defined as initial luspatercept nonresponders. The cumulative mean number of RBC transfusion units or RBC transfusion visits required was estimated using Nelson–Aalen nonparametric estimators with robust variance estimate for each treatment group with a 95% confidence interval.

This post hoc analysis also evaluated the effect of continuing luspatercept treatment beyond the first 24 weeks. Assessment of clinical benefit was conducted up to the data cutoff date of July 1, 2019 (144 weeks of treatment). Clinical benefit indicators included RBC transfusion burden, hemoglobin and serum ferritin levels, and hematologic improvement-erythroid (HI-E) response. HI-E response was assessed per modified IWG 2006 criteria [24] and was defined as a reduction in RBC transfusions of ≥ 4 units/8 weeks for patients with baseline RBC transfusion burden of ≥ 4 units/8 weeks, and an increase in hemoglobin level of ≥ 1.5 g/dL over 8 weeks in the absence of RBC transfusions for patients with baseline RBC transfusion burden of < 4 units/8 weeks.

## Results

### Patient population

A total of 229 patients were enrolled in MEDALIST; of these, 153 were randomized to receive luspatercept and 76 to receive placebo. The baseline characteristics of the patients were well balanced across treatment arms and have been described previously [20]. The number of patients receiving pre- and post-week 25 luspatercept dose levels, following the initial 1.0 mg/kg starting dose, is provided in Table 1. A greater proportion of patients received the maximum dose of luspatercept (1.75 mg/kg; 55%) in the post-week 25 period than in the first 24 weeks (41%). During weeks 25–48, 81% of initial luspatercept nonresponders were treated with the maximum dose of luspatercept (1.75 mg/kg).

**Table 1:** Number of patients receiving luspatercept dose levels before and after week 25

Luspatercept dose level	Before week 25 *N* = 153 *n* (%)	After week 25 *N* = 100 *n* (%)	Last dose received^a^ among initial nonresponders *N* = 68 *n* (%)
0.60 mg/kg	1 (0.7)	1 (1)	0 (0)
0.80 mg/kg	9 (6)	5 (5)	1 (1)
1.00 mg/kg	153 (100)	33 (33)	5 (7)
1.33 mg/kg	102 (67)	36 (36)	7 (10)
1.75 mg/kg	62 (41)	55 (55)	55 (81)

^a^During week 25 through 48

Of the patients treated with luspatercept, 87 (57%) were classified as having low baseline RBC transfusion burden and 66 (43%) as having high baseline RBC transfusion burden and were evaluated for cumulative RBC units and visits over time. In the placebo arm, the proportions of patients with low or high RBC transfusion burden were identical, with 57% (43/76) and 43% (33/76) having low and high baseline RBC transfusion burden, respectively. In the initial 24 weeks of treatment, 58 patients who received luspatercept achieved RBC-TI for ≥ 8 weeks. Of the 95 patients who did not achieve an initial response and were classified as initial luspatercept nonresponders, 68 (72%) continued luspatercept treatment for up to 144 weeks.

### Effect of treatment on number of RBC transfusion units and visits

**Fig. 2:**
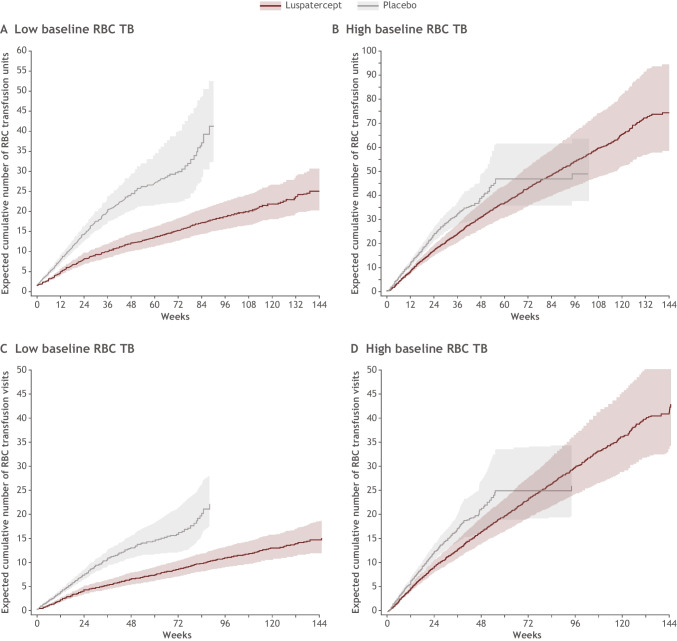
Expected cumulative number of RBC transfusion units (**A**, **B**) and RBC transfusion visits (**C**, **D**) by baseline transfusion burden (low vs high). Low baseline RBC transfusion burden was defined as having < 6 RBC transfusion units over 8 weeks and high baseline RBC transfusion burden was defined as having ≥ 6 RBC transfusion units over 8 weeks. Cumulative mean function estimated using nonparametric method with robust variance estimate. The efficacy cutoff date is defined as the minimum date among death date, study discontinuation date, last dose date + 20, and database cut date. RBC, red blood cell

Overall, during the first 24 weeks of treatment, patients receiving luspatercept had lower mean cumulative RBC transfusion units and RBC transfusion visits than patients treated with placebo, regardless of baseline RBC transfusion burden status (Table 2). This was largely driven by luspatercept responders who had the lowest mean cumulative RBC transfusion units and RBC transfusion visits regardless of baseline RBC transfusion burden category. Expected cumulative number of RBC transfusion units and RBC transfusion visits by baseline transfusion burden is shown in Fig. 2. Beyond week 25, patients with a low baseline RBC transfusion burden who were treated with luspatercept continued to have a lower cumulative number of RBC transfusion units (Fig. 2A) and RBC transfusion visits (Fig. 2C) compared with patients treated with placebo.

**Table 2:** Mean cumulative RBC transfusion units and transfusion visits required during weeks 1–24 of luspatercept treatment by baseline transfusion burden (low^a^ versus high^b^)

Measure	Cumulative mean (95% CI)			
	Luspatercept (*N* = 153)	Luspatercept responders (*N* = 58)	Initial luspatercept nonresponders (*N* = 95)	Placebo (*N* = 76)
Transfusion units				
Low baseline RBC TB	6.8 (5.6–8.4) *n* = 81	2.7 (2.0–3.7) *n* = 49	13.0 (11.4–14.9) *n* = 32	13.2 (11.5–15.2) *n* = 38
High baseline RBC TB	17.2 (15.1–19.6) *n* = 47	3.7 (1.8–7.4) *n* = 6	18.9 (16.9–21.1) *n* = 41	24.2 (21.3–27.4) *n* = 30
Transfusion visits				
Low baseline RBC TB	4.0 (3.3–4.8) *n* = 81	1.7 (1.2–2.2) *n* = 49	7.5 (6.5–8.6) *n* = 32	7.2 (6.3–8.3) *n* = 38
High baseline RBC TB	9.4 (8.3–10.7) *n* = 47	2.0 (1.0–4.0) *n* = 6	10.3 (9.2–11.5) *n* = 41	12.5 (11.0–14.2) *n* = 30

^a^ < 6 units/8 weeks

^b^ ≥ 6 units/8 weeks

Cumulative mean function estimated using non-parametric method with robust variance estimate

*CI*, confidence interval; *N*, number of patients in the intent-to-treat population; *n*, number of patients who completed weeks 1–24 of treatment; *TB*, transfusion burden

### Effect of treatment on RBC transfusion units and visits by luspatercept response at week 25

**Fig. 3:**
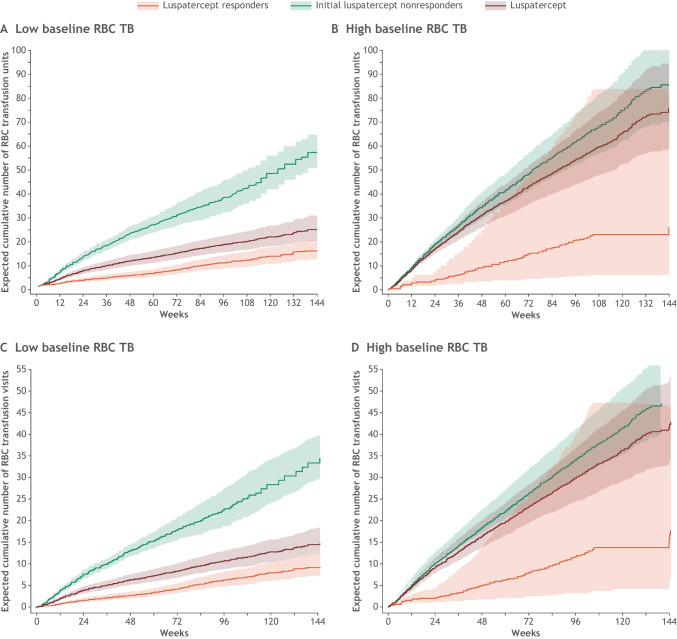
Expected cumulative number of RBC transfusion units (**A**, **B**) and RBC transfusion visits (**C**, **D**) by baseline RBC transfusion burden (low vs high) by luspatercept response at week 25. Low baseline RBC transfusion burden was defined as having < 6 RBC transfusion units over 8 weeks and high baseline RBC transfusion burden was defined as having ≥ 6 RBC transfusion units over 8 weeks. Cumulative mean function estimated using nonparametric method with robust variance estimate. The efficacy cutoff date is defined as the minimum date among death date, study discontinuation date, last dose date + 20, and database cut date. RBC, red blood cell

When categorized by luspatercept response at week 25, patients treated with luspatercept who had responded to treatment by week 25 (achieved RBC-TI for ≥ 8 weeks) continued to show fewer mean cumulative RBC transfusion units required relative to initial luspatercept nonresponders regardless of baseline RBC transfusion burden level (Fig. 3A, B, Table 3). The median follow-up of luspatercept responders was 26 months. Similarly, luspatercept responders at week 25 continued to show a reduction in mean cumulative RBC transfusion visits relative to initial luspatercept nonresponders regardless of baseline RBC transfusion burden level (Fig. 3C, D, Table 3).

**Table 3:** Mean cumulative RBC transfusion units and transfusion visits in luspatercept responders and initial nonresponders by baseline transfusion burden (low^a^ versus high^b^)

Measure	Cumulative mean (95% CI)	
	Luspatercept responders (*N* = 58)	Initial luspatercept nonresponders (*N* = 95)
RBC transfusion units		
Low baseline RBC TB		
48 weeks	4.7 (3.5–6.3) *n* = 43	22.8 (20.1–25.8) *n* = 15
144 weeks	15.2 (11.5–20.0) *n* = 5	57.1 (50.5–64.6) *n* = 1
High baseline RBC TB		
48 weeks	9.3 (3.5–25.0) *n* = 6	34.7 (29.6–40.7) *n* = 21
144 weeks	26.1 (8.3–81.9) *n* = 1	85.6 (70.1–104.5) *n* = 1
RBC transfusion visits		
Low baseline RBC TB		
48 weeks	2.8 (2.1–3.7) *n* = 43	13.1 (11.8–14.6) *n* = 15
144 weeks	9.3 (7.2–12.2) *n* = 5	33.4 (28.8–38.9) *n* = 1
High baseline RBC TB		
48 weeks	5.0 (1.8–13.5) *n* = 6	18.5 (16.1–21.2) *n* = 21
144 weeks	15.5 (5.2–46.3) *n* = 1	47.0 (39.1–56.5) *n* = 1

^a^ < 6 units/8 weeks

^b^ ≥ 6 units/8 weeks

Cumulative mean function estimated using non-parametric method with robust variance estimate

*CI*, confidence interval; *N*, number of patients in the intent-to-treat population; *n*, number of patients with RBC transfusion data up to timepoint; *TB*, transfusion burden

### *Clinical outcomes in patients who did not achieve RBC-TI at* ≥ *8 weeks but continued luspatercept treatment beyond week 25*

#### Weeks 25–48 of the extension phase

Of the 68 patients who were initial luspatercept nonresponders (by HI-E) at week 25 and continued treatment, 11 patients (16%) achieved RBC-TI for ≥ 8 weeks during weeks 25–48 of the extension phase. Of these, 3 patients were RBC-TI for ≥ 16 weeks (Online Resource 1 Figure). The median time to achieving RBC-TI for ≥ 8 weeks was 5 months from the beginning of week 25 (i.e., approximately 11 months from the first dose in the primary treatment phase, weeks 1–24).

During weeks 25–48, 18 (26%) of the 68 initial luspatercept nonresponders had a reduced RBC transfusion burden relative to baseline. In this group, the mean change (standard deviation (SD)) from baseline in the RBC units transfused was − 3.1 units (6.51) during the first 24 weeks of treatment, rising to − 6.5 units (6.31) during weeks 25–48. In initial luspatercept nonresponders with a full 48 weeks of available treatment data (*n* = 36), the mean change (SD) from baseline in RBC units transfused was − 1.3 units (7.98) over a 24-week treatment period. Serum ferritin levels relative to baseline were reduced during weeks 25–48 in 30 (44%) of the 68 initial luspatercept nonresponders. By week 48, the serum ferritin level in patients who had a baseline serum ferritin level of ≥ 1000 µg/L had dropped to < 1000 µg/L in 7 (18%) of 39 patients.

**Fig. 4:**
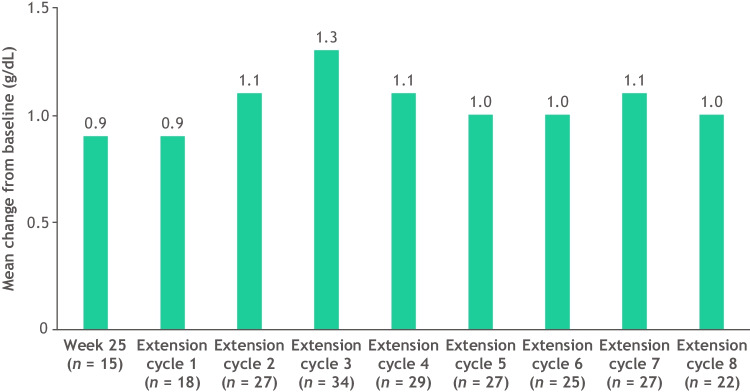
Mean change from baseline in hemoglobin levels, weeks 25–48, in initial luspatercept nonresponders. Number of patients varies as not every patient underwent hemoglobin assessment each week

As a descriptive analysis, the mean change from baseline in hemoglobin levels from weeks 25 to 48 for initial luspatercept nonresponders is shown in Fig. 4 (for patients who had been assessed for hemoglobin at the time point). The greatest mean increase from baseline was 1.3 g/dL, which occurred at extension phase cycle 3 (beyond week 25) (Fig. 4).

**Fig. 5:**
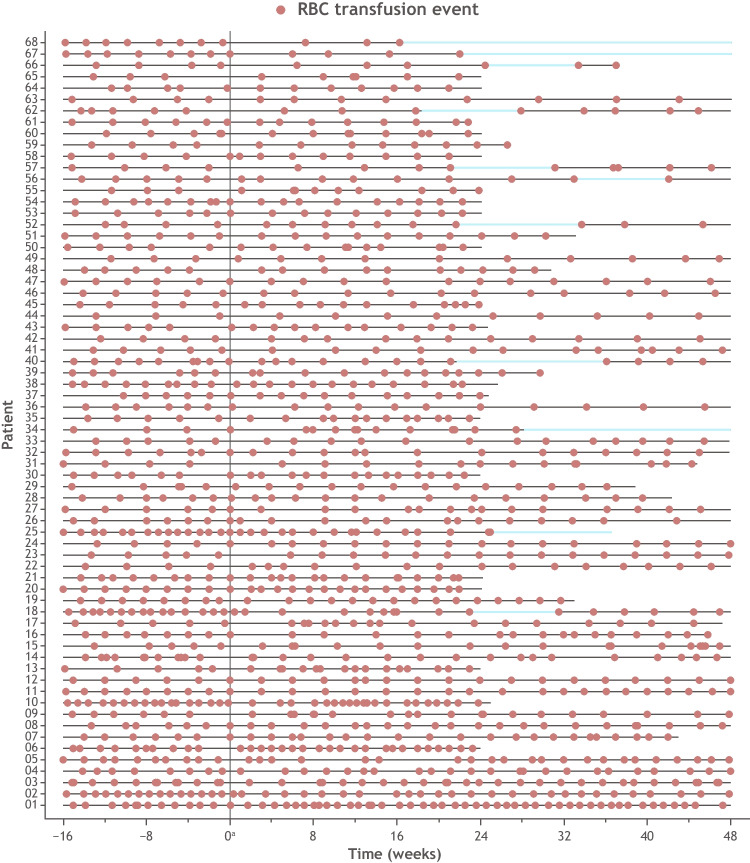
RBC transfusion events in initial luspatercept nonresponders pre- and post-initiation of luspatercept treatment. Periods of response are denoted by blue lines. ^a^Start of luspatercept treatment. RBC, red blood cell

RBC transfusion events for the 68 initial luspatercept nonresponders during the 16 weeks prior to and 48 weeks after the start of luspatercept treatment are shown in Fig. 5. This includes the 11 (16%) patients who continued luspatercept beyond week 25 and ultimately achieved RBC-TI for ≥ 8 weeks despite being classified as initial luspatercept nonresponders at week 25.

#### Weeks 1–48 and during the entire treatment period

**Fig. 6:**
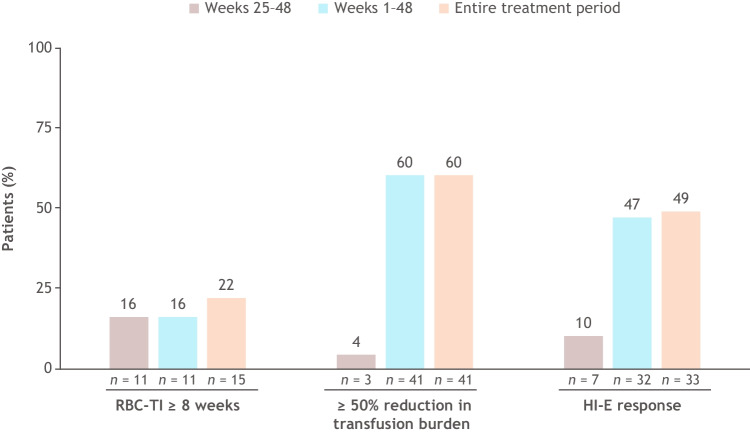
Response indicators across analysis periods. Evaluated in patients who did not achieve RBC-TI for ≥ 8 weeks by week 25 but continued luspatercept treatment (*n* = 68). HI-E, hematologic improvement-erythroid; RBC-TI, red blood cell transfusion independence

Clinical benefit as assessed with the response indicators RBC-TI for ≥ 8 weeks, ≥ 50% reduction in transfusion burden, and HI-E response is shown in Fig. 6 across analysis periods during treatment. During weeks 1–48, of the 68 patients who were initial luspatercept nonresponders at week 25, 41 (60%) had achieved a ≥ 50% reduction in RBC transfusion burden for ≥ 8 weeks from baseline and 32 (47%) had achieved an HI-E response. Beyond 48 weeks and through 144 weeks, 4 additional patients achieved RBC-TI for ≥ 8 weeks and 1 additional patient achieved an HI-E response.

## Discussion

In the MEDALIST study, luspatercept demonstrated a consistent benefit in reducing RBC transfusion burden within the first 24 weeks of treatment among patients with LR-MDS [20]. In this analysis, patients who had responded by week 25 of MEDALIST continued to show a benefit of luspatercept treatment in terms of lower cumulative RBC transfusion units and RBC transfusion visits, regardless of baseline RBC transfusion burden levels, over a median follow-up of 26 months. A considerable proportion of the 68 patients who did not achieve RBC-TI for ≥ 8 weeks at week 25 and continued on luspatercept experienced clinical benefit during the entire treatment period. Clinical benefits, observed through 144 weeks in initial luspatercept nonresponders, included RBC-TI for ≥ 8 weeks in 22% of patients, ≥ 50% reduction in RBC transfusion burden in 60%, an HI-E response in 49%, improved hemoglobin levels of up to a mean gain of 1.3 g/dL from baseline, and reduced serum ferritin compared with baseline in 44%. For most (81%) initial luspatercept nonresponders, luspatercept was titrated to the maximum dose level (1.75 mg/kg) during weeks 25–48 for these clinical benefits to be achieved.

The findings of this analysis have value in the real-world clinical setting where luspatercept can lower transfusion burden and continue to offer clinical benefits beyond 25 weeks of treatment. This analysis has revealed a lower cumulative transfusion burden in terms of the number of RBC transfusion units and visits with continued luspatercept treatment and has shown that transfusion burden among initial luspatercept nonresponders can be improved by extending treatment. Continued luspatercept treatment beyond 25 weeks may provide RBC-TI and hemoglobin improvement for patients who require additional time to achieve a response indicative of clinical benefit.

Achieving transfusion independence in LR-MDS is an important treatment goal given the negative impact of RBC transfusions on OS and leukemia-free survival in patients with LR-MDS [6, 11–14], the humanistic burden of chronic anemia in patients [14, 16], and the risk of iron overload [8]. Our findings have shown that extending treatment with luspatercept beyond 25 weeks may be of benefit for patients with LR-MDS who were initially less responsive.

According to the package insert, treatment with luspatercept should be discontinued if a patient does not experience a decrease in transfusion burden after 9 weeks of treatment at the maximum dose level or in cases of unacceptable toxicity [21, 22]. In the MEDALIST study, decisions regarding continuation of treatment in patients not meeting stringent parameters indicating response were made by individual clinicians based on their assessment of clinical benefit in terms of reductions in RBC transfusion burden compared with baseline; increased hemoglobin and decreased serum ferritin levels compared with baseline; and HI-E response. In the real-world setting, clinicians’ assessment of improvement in clinical features and, critically, how their patients perceive the balance between burden of treatment and benefit, in terms of reduced anemia and transfusion burden, should guide decisions regarding continuation of treatment. In line with prescribing guidelines, most of those patients going on to achieve benefit after week 25 in the MEDALIST study did so on the highest dose of luspatercept, making maximal titration, when tolerated, a key consideration before discontinuation.

A strength of this analysis is that the patient-level data were obtained from a large multinational phase 3 randomized, placebo-controlled trial. Limitations include its nature as a post hoc analysis focused on a subgroup of patients not attaining a response, resulting in the loss of statistical inference attainable from a randomized population. Therefore, these results should be confirmed in future prospective analyses [25]. In addition, the substantial drop-out in the placebo group beyond week 25 may have minimized the robust comparison between groups during the longer follow-up period.

In conclusion, our findings suggest that continuing luspatercept treatment beyond 25 weeks may provide clinical benefit for a meaningful proportion of patients with LR-MDS with ring sideroblasts who otherwise have limited treatment options. These findings may inform clinical practice treatment decisions with regard to the timing of clinical benefit, the relevance of increasing the dose level of luspatercept in the absence of an initial response, and the consideration of luspatercept treatment for patients with high or low pretreatment RBC transfusion burden to facilitate a rational identification of the patients most likely to benefit.

## Supplementary Information

Below is the link to the electronic supplementary material.

Supplementary file1 (DOCX 103 KB)

## Data Availability

The BMS policy on data sharing may be found at https://www.bms.com/researchers-and-partners/independent-research/data-sharing-request-process.html.
