# Supplementary material for: Improved benefit of continuing luspatercept therapy: sub-analysis of patients with lower-risk MDS in the MEDALIST study
Source: Ann Hematol. 2023 Jan 13;102(2):311–21. doi: 10.1007/s00277-022-05071-8 (PMC9889415; doi:10.1007/s00277-022-05071-8)

**Online Resource 1 Figure** Design and patient disposition: post-hoc analysis of the MEDALIST trial. RBC-TI, red blood cell transfusion independence


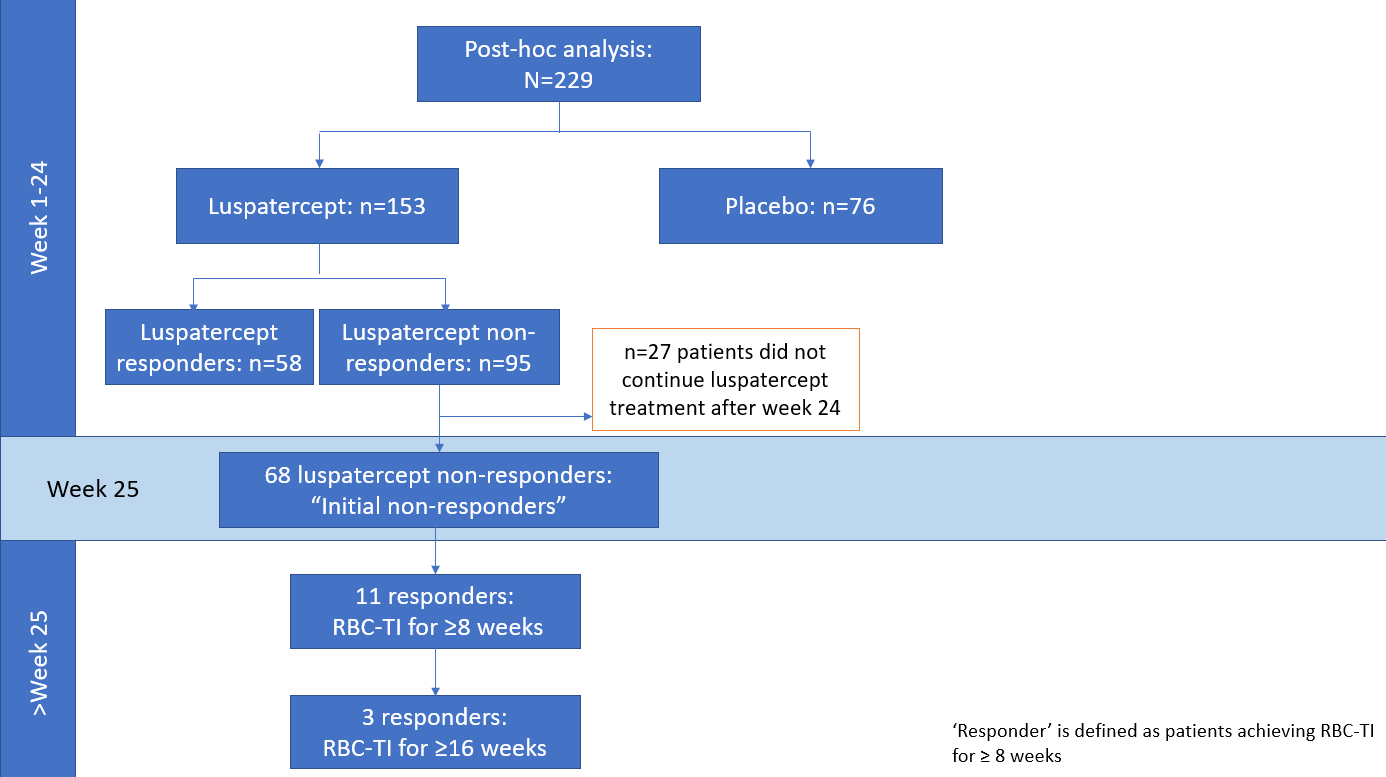

Supplement: Supplementary file 1 — Supplementary file1 (DOCX 103 KB) [file 277_2022_5071_MOESM1_ESM.docx]
